# Point-of-care testing (POCT) for HIV/STI targeting MSM in regional Australia at community ‘beat’ locations

**DOI:** 10.1186/s12913-019-3899-2

**Published:** 2019-02-02

**Authors:** Amy B. Mullens, Josh Duyker, Charlotte Brownlow, Jime Lemoire, Kirstie Daken, Jeff Gow

**Affiliations:** 1 School of Psychology & Counselling, Institute for Resilient Regions, University of Southern Queensland, 11 Salisbury Rd, Ipswich, QLD 4305 Australia; 2 School of Psychology & Counselling, Queensland University of Technology, Kelvin Grove, QLD 4059 Australia; 3 Queensland Positive People, 21 Manilla St, East Brisbane, QLD 4169 Australia; 4 School of Psychology & Counselling, Institute for Resilient Regions, University of Southern Queensland, Main St, Toowoomba, QLD 4350 Australia; 5 School of Commerce, Institute for Resilient Regions, University of Southern Queensland, Main St, Toowoomba, QLD 4350 Australia; 6 School of Accounting, Economics and Finance, University of KwaZulu-Natal, Durban, 4000 South Africa

## Abstract

**Background:**

Innovative health promotion strategies are needed to improve access to HIV testing among regional people in Australia, particularly for men who have sex with men (MSM). This project aimed to establish proof of concept for point-of-care-testing (POCT) via a mobile van clinic at community ‘beat’ locations. Surveys evaluated client satisfaction, characteristics and testing preferences among ‘early adopters’. Sequential mixed-methods approach was used which included secondary qualitative analysis of field notes written by peer-testers (i.e., trained lay providers from the key population being targeted; to extend the contextualise the pilot evaluation), documenting barriers/facilitators and innovations, per action research and to guide recommendations for future health promotion initiatives.

**Methods:**

A POCT ‘proof of concept’ project (2, 3-hourly sessions/week; 20 weeks) was delivered in a regional town by peer-testers using a mobile clinic van, recruited by geosocial ‘apps’ targeting MSM. Clients completed surveys regarding demographics, and testing satisfaction, frequency and preferences. Peer-testers completed detailed field notes for each session including client characteristics and impressions, salient events, concerns and recommendations.

**Results:**

The program resulted in 34 online health promotion conversations with MSM and 34 POCT tests (19 HIV, 15 Syphilis; 18 unique client visits; 17 identified as MSM, with 1 heterosexual female. Rates of satisfaction among early adopters of POCT was high. Analysis of field notes revealed three major themes: 1) Practical challenges; 2) Barriers to engagement; and 3) Recruitment method/project promotion.

**Conclusions:**

Amongst early adopters satisfaction was high, with 47% of clients reported infrequent testing (over 12 months ago) or having ‘never tested’. No tests were reactive. Challenges associated with this health promotion initiative and recommendations for future HIV testing promotion and programs were outlined.

**Electronic supplementary material:**

The online version of this article (10.1186/s12913-019-3899-2) contains supplementary material, which is available to authorized users.

## Background

People living in regional and rural areas in Australia experience reduced access to sexual health services, compared to urban dwellers. Those accessing testing services in regional or rural areas may face actual and perceived barriers, such as concerns regarding confidentiality and privacy [1]. Human Immunodeficiency Virus (HIV) stigma may be a salient barrier [1]. These difficulties contribute to infrequent rates of testing and lead to undiagnosed cases of HIV and sexually transmitted infections (STI) among those at greater risk, men who have sex with men (MSM); and late diagnoses contribute to subsequent onward transmission and poorer health outcomes [2, 3].

In 2015, approximately one-third of new HIV diagnoses in Queensland (Australia) occurred outside of metropolitan centres [4]. The prevalence of HIV in rural, regional and remote Queensland is difficult to ascertain [5]. Innovative services which promote opportunistic or early detection of HIV/STI are crucial to reduce/prevent HIV-related health disparities [6]; and increasing access and uptake of voluntary testing through point-of-care-testing (POCT) in community-based settings among key rural and remote populations [7].

Community-based, peer-led HIV/STI testing models have been demonstrated to be beneficial for individuals who have difficulty accessing mainstream health services [8–11]. Mobile clinic interventions utilising POCT testing have been an acceptable and feasible way to engage MSM in urban areas [12–15]. However, research investigating POCT among regional MSM is very limited [16]. The non-governmental organisation (NGO), Queensland Positive People (QPP), through its RAPID clinic and in collaboration with the University of Southern Queensland (USQ) developed a POCT mobile intervention which was piloted in a regional centre in South East Queensland at community ‘beat locations’ during late 2016 and early 2017 [17].

This intitative aimed to reduce barriers to HIV/Syphilis testing among ‘at risk’ populations, predominently regional MSM (who may be reluctant to engage with mainstream health services in regional towns), to promote testing frequency and establish ‘proof of concept’—from the perspecitves of clients and peer-testers. Participants were also offered the opportunity to engage with peer-testers in a safe, non-judgemental environment, to discuss health promotion and risk reduction strategies (e.g. pre-exposure prophylaxis [PrEP], post-exposure prophylaxis [PEP]), and options for future HIV testing.

Using a sequential mixed-methods approach, this study will: 1) Establish proof of concept of a mobile clinic van intervention as a method to engage individuals in regional Queensland with anonymous HIV/STI POCT, particularly focussing on MSM as a key population group; 2) Describe characteristics of early adopters of this service; and 3) Describe facilitators and barriers to implementation of this time-limited program. In-depth qualitative analyses of peer-tester field notes provide rich insights regarding the challenges faced and innovations developed per ‘action research’. Recommendations for future regional sexual health promotion programs will be made.

## Methods

### Project overview

A regional town in Queensland (the regional town most proximal to the RAPID metropolitan centre in Southeast Queensland) was selected as the pilot site, as studies indicated that syphilis rates in this community had recently significantly increased [18]. Further, this region had eleven diagnoses of HIV in 2016, with an estimated HIV prevalence rate of 4.0 per 100, 000, the highest of any regional area in the state [19]. This situation combined with limited access to sexual health testing for MSM were a major concern indicating the possibility for trialing an innovative HIV/STI testing approach. There is one dedicated site for STI/HIV screening and treatment in the region, which is located within a government-based sexual health service and large hospital. This region is anecdotally regarded as a ‘conservative area’ [20] with no known ‘sex on premises venue’, however with a reportedly active ‘beat’ scene [17].

### Formative assessment

Consultative meetings commenced in November 2015 with a range of community members, service providers and public health stakeholders regarding potential need, benefits and challenges of the proposed POCT program. The lesbian, gay, bisexual, transgender, queer and intersex (LGBTQI) police liaison officer and local council officers were engaged to assist in determining the most suitable beat locations and testing times [17, 21]. The site was chosen due to extensive community engagement vie the HIV Foundation Queensland ‘RAPID Roadshow’ 2015 [20] during formative assessment, prior to the current project. From a practical sense, it was chosen as it was a regional area that was easily accessible from Brisbane (state capital). Thus, this study built upon previous work conducted in the region with key population stakeholder engagement for improved relevancy and efficiency of the public response for health promotion [21]. Ethical approval was gained through the university Human Research Ethics Committee (H16REA117).

### Recruitment and online engagement

Recruitment was achieved through advertising and profiles on geo-social networking applications and websites (Grindr and Squirt, as MSM were the target group for the intervention—however others seeking testing for actual or perceived risk would not be excluded, which is consistent with the RAPID philosophy). RAPID had active profiles on both sites which was moderated by peer-testers (who were paid employees of RAPID and identified as PLWH and/or MSM), and the POCT program was advertised via RAPID website.

### Testing philosophy

As a peer-based organisation, Queensland Positive People (QPP) provides HIV testing through a respectful and innovative community-based client-centred approach. The focus is on supporting the early identification and diagnosis of those living with HIV in the community and provide early treatment, improve health outcomes, enhance connections with people living with HIV (PLHIV), and reduce the onward transmission of HIV. POCT is targeted towards increasing testing among MSM with the trained staff members who provide the testing being from the affected community, express a respectful understanding of diverse sexual communities, demonstrate progressive understanding of combination prevention strategies and are able to provide a safe peer environment to support informed choice for those who choose not to use condoms in the treatment era: undectable and PrEP [22].

### Mobile clinic POCT

Between 15/09/2016 and 05/02/2017, RAPID peer-testers provided HIV/Syphilis POCT and health promotion via mobile clinic van. One evening and one morning/midday clinic for 3 hours each was provided weekly. The van was parked near known ‘beats’ and two sites were selected due to feedback from key informants in the area, their popularity/consistent activity on the message boards of Squirt and their ability to discreetly park a mobile clinic van without interfering with beat activity or drawing external attention to the area. The mobile clinic van was staffed by peer-testers who provided POCT using the Uni Gold-HIV (Trinity Biotech, Wicklow, Ireland) and Alere HIV Combo and Alere Determine Syphilis (Alere Medical, Sinnamon Park, Australia) tests. The screening/eligibility process was broad and including being over 18 years old and having a real or perceived risk of HIV/STIs. Although recruitment was focused on MSM, a HIV test was available for anyone who requested one. The screening process included a brief conversation with the participant consistent with the RAPID philosophy of ‘receiving a test without the Q&A’ (with no further screening questions often experienced within mainstream services; which presents a barrier to engagement to many MSM [23]). A brief screening process can also help to reduce stigma and potential barriers to testing [22]. Participants (100% of those attending the POCT consented to participating in the research) provided verbal consent prior to testing and completion of a post-test questionnaire, and information discussions were held with participants regarding their last test as part of the pre-test consultation (however, was not collected in the research protocol for evaluation of proof of concept) (Additional file 1). The post-test questionnaire used in this study was created by Queensland Positive People as per the organisation’s standard evaluation procedure with additional items included that were relevant to the current project and research questions.

### Field notes

Peer-tester field notes on their experiences described in detail the circumstances of program delivery. These included details on the location and progress of the clinic, as well as reflections and detailed information from participants and described ways the pilot could be improved to provide a more comfortable testing environment, as well as circumstances affecting the number of participants (e.g. locations during rain).

### Qualitative analysis of field notes

Data from the field notes reflected key factors associated with health promotion and client engagement, and challenges/solutions with project management. These notes were collated and analysed using principles of thematic analysis [24]. This analysis required re-reading through the quotes independently multiple times in order to identify common themes. These were synthesised into dominant themes following researcher discussions. This information was reviewed by an additional reviewer with only minor adjustments subsequently made. This ensured a rigorous and accurate interpretation of the field note data.

## Results

### Online engagement and recruitment

During the 18 weeks of the project there were 34 meaningful online health promotion conversations with MSM documented via the apps [25], and including the following topics: other RAPID testing locations (*n* = 15), sexual health queries (*n* = 6); testing locations (*n* = 5) [inside (*n* = 2) and outside of region (*n* = 3)], anonymity of testing (*n* = 5), PrEP (*n* = 2), and HIV self-testing (*n* = 1). More than half were recruited via Grindr while a minority were previous RAPID testers or recruited via ‘word of mouth’ or Squirt.

### POCT testing and characteristics of early adopters and testing

Nineteen HIV and 15 syphilis tests were conducted (*n* = 18 people, 1 person tested twice). The majority of clients were men (*n* = 17; 1 female); and identified as gay (*n* = 14), bisexual (*n* = 3) or heterosexual (*n* = 1). Ages ranged from 18 to 67 years, with a median age of 40. No participants identified as Aboriginal or Torres Strait Islander, and no participants reported injecting drug use. The majority (*n* = 17) of participants were born in Australia and all reported having a Medicare card. All tests were non-reactive. Almost half of MSM clients attending the POCT had a discussion with a peer-tester on Grindr or Squirt before testing; and typically, the length of time from initial contact with the peer-tester to testing was about 4–6 weeks.

### Questionnaire: Satisfaction, testing frequency and preferences

Overall, the data from the post-testing questionnaire (response rate 95%; Table 1) indicated high satisfaction and acceptability of the service among the limited number of ‘early adopters’ (based on questions within RAPID testing service minimum dataset [22]; as well as scoping potential interest for future regional home-testing initiatives). The majority of participants (78%, *n* = 14) strongly agreed or agreed that the mobile clinic van was an acceptable POCT method. Participants reported that they found this service acceptable as it was “discrete”, “because it is easy to get to”, “it just seems unintrusive”. The majority of participants (78%, *n* = 14) also responded that they would be happy to refer a friend to the testing facility 89% (*n* = 16) of participants reported that their testing frequency would increase if the mobile clinic came regularly. Some participants preferred using home testing methods, while 50% (*n* = 8) of participants responded either strongly agreed or agreed that they found it easier to test from a mobile van clinic located near a ‘beat’.

**Table 1:** Survey Item Responses regarding Testing

	Strongly Agree	Agree	Unsure	Disagree	Strongly Disagree
Satisfaction					
I would be happy to refer a friend for HIV/sexual health testing via an incentive coupon:	10	4	3	1	
Community HIV testing from a mobile clinic van is an acceptable HIV testing method:	14	4			
Frequency					
A peer-led, community based testing service like the RAPID mobile van clinic would increase my HIV testing frequency:	13	3	1	1	
I would have had a HIV test regardless of whether the mobile clinic van was available:	4	4	9	1	
If the mobile clinic van came regularly, my HIV/sexual health testing frequency would increase:	12	4	1	1	
Testing Preferences					
I find it easier to test for HIV from a mobile clinic van located near a 'beat‘:	5	4	6	3	
I would prefer to test for HIV anonymously:	8	2	5	1	
I would be willing to use a HIV home testing kit after a referral from the mobile clinic van:	8	6	2	2	
I would be willing to use a HIV home testing kit in the future:	7	7	3	1	
I would be willing to pay for a HIV home testing kit:	6	7	2		1

Table 1 displays the responses to survey items as grouped by general themes of satisfaction, frequency and testing preferences (based on existing RAPID post-testing evaluation in other settings). The findings are intended to be descriptive in nature and capture a ‘snapshot’ of general beliefs and perceptions regarding testing, and not intended to substantiate more formal constructs per se.

### Qualitative analysis of peer-tester field notes

The thematic analysis of field notes with a specific focus on barrier/facilitators and innovations (per action research) to POCT vie mobile clinic van in a regional area targeting MSM identified three dominant themes: *Practical challenges*, *Barriers to engagement*, and *Project effectiveness and future directions*.

#### Theme 1: Practical challenges

Practical barriers to engagement were perceived by the peer-testers, including the nature of the RAPID POCT equipment and the need for and assurance of their personal safety.

### The right ‘look’ and equipment

A key challenge was regarding reliable and appropriate equipment and visibility was cited as being of upmost importance in the engagement of clients, for example:

We were sitting in an unmarked truck located down the end of a one-way street. We had 3 cars essentially do a drive by, maybe if we had signs on the truck they may have stopped in. 14th September

Similarly, frustration was expressed regarding the lack of reliable equipment with missed opportunities (e.g., flat tyre). Once signage was created (removable magnets), a much more approachable service was presented to potential clients.

Appropriate equipment was, therefore, considered a central issue in creating the appropriate ‘look’ in order to engage potential clients for testing given the often ‘hard-to-reach’ nature of the target group.

### Personal safety of peer-testers and the need for safe engagement

The personal safety of the peer-testers was strongly held during this project. The testing van was in operation at night, often in isolated places, sometimes in an unfamiliar area. One particular incident highlighted the need to review security protocols for the peer-testers:

As the cars had moved, we decided to move the truck to our usual spot. After we had moved there were at least 3 utes that did drive-bys. As we had the RAPID magnet on the back of the truck, one couple stopped to ask what RAPID was. One of the trucks that did a drive by parked down the road and one other shortly joined them. One truck with rowdy teenagers hung around for a while and drove off when ‘Tester 3’ sounded the alarm. After a little while, more cars with rowdy teenagers pulled up close to us. ‘Tester 3’ continued to sound the alarm. When it was 5 cars filled with drunk boys, we decided to pull the pin for safety. 12th October

This scenario demonstrates the uncertain situations the peer-testers often experienced. The need for private, isolated testing places in order to attract potential clients introduced additional risks. This particular incident led the team to review and further develop security protocols. For example:

- One individual needs to have self-defence training if beat walking is to be undertaken.
- If we walk around, we will only do it in pairs and only walk where there is plenty of light.
- No more external signage on the truck. We are to use nonverbal language to engage with people, like waving, and only to single occupancy vehicles. 12th October

Thus, a delicate balance needed to be found between promoting the ‘right look’ in order to attract potential clients but not being too visible in order to not attract unwanted attention. This delicate balance is further explored in the second theme, which focuses on perceived barriers to engagement.

#### Theme 2: Barriers to engagement.

The peer-testers extensively reflected on other perceived barriers to engaging potential clients, notably the importance of understanding the specific community culture, issues of trust and anonymity, and the negotiation of professional boundaries.

### Importance of understanding the specific community culture

It was important to understand the local culture of the testing sites as well as the online culture and use of social media as a way to engage with clients:

I checked the Squirt website and the [*name] beat has been actively used over the past few days. It seems that our presence is known on Squirt, but our location isn’t. In a way, this is a good thing because if guys knew where we were, they might not turn up to the beat. 21st September

Selecting an appropriate location for testing was therefore an ongoing challenge for the team as reflected on by ‘*peer-tester 1’:

After my cruising experience at the toilet block last week, I decided to move into the big bush that we park the truck next to. It was quite open and spacious on the inside. Although there was no evidence of this space being used as a beat, the police liaison officer said that a lot of activity happen in the bushes at night time. This would be a prime location for hook ups. 22nd September

The local culture of the town was also a central issue specifically the cohesion of MSM within the town and the choice to be tested in locations outside of where they live. For example:

A guy on Squirt was asking about testing on the Gold Coast and at [*clinic name]. Maybe it is quite possible that some South-East Queensland MSM travel to Brisbane and the Gold Coast for testing? 15th September

I am unsure if respondent-driven sampling (RDS) is going to be acceptable in this community. It seems very fragmented and people do not want to connect, and if they do connect, they want to do so anonymously. 21st September

These quotes indicate an additional challenge—the need to understand local MSM culture and the need for assurance of confidentiality in a marginalised community in a conservative regional town.

### The importance of trust and anonymity

The development and maintenance of trust within the target group was considered paramount for the successful engagement of clients, and issues of confidentiality and anonymity were cited by several potential clients. For example:

Some guys are interested in the testing, but have voiced concerns around anonymity and the tests accuracy. 14th September

In addition, several queries were made on social media platforms to clarify the actual anonymity of the RAPID mobile service. Issues of maintaining anonymity amongst peers was voiced as a pivotal concern, as was the potential for local professionals to ‘gossip’. For example:

Two tests were completed today that reinforce the value of this project. The first client was a bisexual male with MSM risk of HIV. He disclosed not wanting to test at [the local clinic] because “he worked in the area and this was just too small a town”. 8th December

Shortly after arriving, a woman approached us for HIV testing. She had had some potential occupational risk for HIV transmission and disclosed that because of [place name] being a ‘small’ town and people gossiping that she wanted to test with someone from out of town. 24th November

While concerns about anonymity could be discussed with clients, the small community was perceived to heighten the risks for potential personal disclosure by others. However, what was interesting from the examples above was that the mobile service may be attractive, not only to discrete MSM in regional towns, but also importantly for other communities and individuals who may be at risk of HIV who share similar stigma and challenges to disclosure.

### Negotiating ‘relationship boundaries’

The second core issue to manage was regarding boundaries for themselves as peer-testers, when they may be approached by beat users who may be interested in them socially/sexually. The need to reinforce their professional roles was strongly held. Several examples of ‘misconceptions’ by potential clients as to the purpose of their presence were evident, for example:

There was one truck that parked next to us and a guy just sat there looking at us for a while before leaving. I needed to go to the bathroom, so I went across the road to the cruisy toilet block. As I was entering the bathroom, he was exiting. The next minute he was standing next to me at the trough – I got cruised! I left quickly after that, but it made me think that we are in a good location. Had I more courage, I would have struck up a conversation with him and encouraged him to get tested, but I didn’t think it would have been appropriate. 15th September

A guy pulled up next to us and had a chat with John. John informed him of what we were doing, and they had a brief chat before he sat down and pulled out his dick. John got a bit awkward and came back in the truck. Before he had the chance to give him some information and resources, the guy got back in his car and drove away. 6th October

Such incidents prompted the team to consider what strategies might be appropriate for engaging clients. Non-verbal cues were considered to be most effective, however these led to mixed interpretations at times, for example:

Today we are also exploring the non-verbal cues that we can use to engage with guys, but not be too invasive. Waving seems to work some of the time, but it hard to tell if we are engaging with the right people. Prolonged eye contact with people turning back to look again seems to be working well. Guys aren’t coming back to talk to us, but we are definitely engaging with them using this technique. We are clearly identified as health workers (wearing our RAPID shirts) and it is not our intention to manipulate guys into thinking we are cruising them, it’s just a way for us to break the ice. 13th October

Engagement continued to be a challenge throughout the project, however incidents such as these led to the development of nonverbal protocols for initiating engagement in a professional way. Nonverbal cues were acknowledged as having the potential to be misread by others, and therefore needed to be carefully considered to reduce the likelihood of sending ‘mixed messages’:

‘*Peer-tester 3’ and I decided that instead of focusing on ‘non-verbal’ cues, we just need to be visible and approachable. We discussed what exactly it is that makes us visible and approachable, and funnily enough, a lot of it is non-verbal cues. I just think that if we do the eye contact with guys, we are just going to be told that we are cruising guys. 26th October

The negotiation of relationships between the peer-testers and clients was of central concern, and required much consideration of safeguarding trust and anonymity. The final theme combines points raised in the previous two themes to further refine the recruitment method and promoting the service to meet the needs of future clients.

#### Theme 3: Project effectiveness and future directions.

This specifically drew upon discussions regarding current meaningful engagement with health promotion initiatives, concerns and suggestions regarding the current project, and recommendations for the future.

### Meaningful engagement with the health promotion project

The use of social media was seen as being very effective in engaging individuals for testing. These platforms were used successfully for promoting the service and responding to clients’ concerns and questions. For example:

We have only had two chats tonight – one on Grindr and one on Squirt. Both have come in to test! I greeted both clients as they have approached the truck and that seems to have put them at ease straight away. It seems the advertising has been good at raising awareness, but the conversations on Grindr and Squirt is what is actually getting clients in to test.

The first client we tested tonight was very sweet and provided some good feedback about how we made him feel comfortable to do his very first test. He will come back for another test before we finish up here at this location. 21st September

I also received a message on Locanto (online classifieds website) from the ad I posted two weeks ago with someone asking about how to access our testing. 6th October

We had a few chats on Grindr – most wanted to know about times and locations and about the testing itself. 2nd November

It was further hoped that such positive engagements, as a result of social media, would lead to ‘word of mouth’ recommendations. For example:

The guy who I chatted to from Grindr came in to get tested today. He didn’t seem to have any barriers to [*local testing site] except that he travelled a lot for work. He also has been to RAPID before … He seemed impressed with the service and said that it was invaluable. 10th November

### Concerns and suggestions for the current project

There were other reflections on the current project and suggestions that social media engagement should not be at the expense of non-social media users. For example:

My encounter with the older gentleman in the bathroom made me think of the limitation we discussed in regards to online sampling recruiting: if we only advertise and recruit online, we are going to miss out on all the guys who don’t use the internet/apps to meet other guys. 15th September

Such reflections prompted discussions regarding the need to broaden the recruitment strategies, especially when traffic was slow via social media platforms:

If respondent driving sampling isn’t going to work…and the discrete targeted advertising isn’t getting punters in, what else can we do to get people in to come to test? 28th September

The need to more carefully engage with the specific needs of the local community, who may or may not be social media users, was explored in order to strike a balance between visibility, and protection of anonymity and respect for individuals:

Another suggestion was to put some cards in the toilet blocks of known beats. This could be a good idea, but I would be worried that council will just throw them out or that they could get into wrong hands and create the wrong kind of attention (remembering that [town] is a very conservative area.) 29th September

The strategy of placing cards in toilet blocks on known beats during the hours of RAPID service was explored with some success, at least in terms of raising awareness:

‘*Peer-tester 3’ left the testing cards in the toilet block, one on the urinal and one on the sharps box. They have been taken up by potential clients. We are considering doing a beat run each trip and dropping cards at all of the active beats in the hope of increasing recruitment. 26th October

## Discussion

The notion of conducting a recurring discreet mobile clinic van testing service utilising peer test facilitators providing HIV/Syphilis POCT to at-risk, MSM in a regional area has been a unique achievement, and a first of its kind within Australia. However, the use of POCT in regional and rural settings in other countries within other key populations [26, 27]; and among MSM in other community settings within Australia [7, 14, 28] is well established.

‘Hard to reach’ groups (like regional/rural MSM) are harder to engage in health services, requiring more time to develop trust and requiring more resource-intensive support to address specific health needs [29–32]. Thus, this new mobile POCT approach needed to gain the trust of the community, especially as rates of stigma, discrimination and marginalisation of MSM are higher in regional/rural areas and among further stigmatised sub-groups [33]; and it’s been demonstrated elsewhere that MSM attending non-conventional HIV testing locations may have higher rates of condomless sex than those attending conventional sites (e.g., general practitioners [GP]), and represent a key target group for health promotion and HIV screening (e.g., self-testing; [34]). Processes adopted during the formative assessment phases to engage with key community members and organisations to ‘share priorities’ is consistent with best practice in health promotion [21].

### Project summary

The acceptability of the mobile van clinic intervention was high among the limited sample of ‘early adopters’. It was expected that recruitment would be slow due to heightened stigma regarding HIV/STI testing in a regional community, particularly among the target group (MSM); as well as challenges associated with similar health promotion attempts [35, 36]. There was a large proportion of irregular and first-time peer-testers who were part of the target group (MSM); which was evidenced by peer-tester notes; although not part of the research protocol per se due to non-invasive RAPID philosophy. Although the intervention was intended to be discreet and anonymous, the mobile van clinic model is unique and may have been viewed by some MSM as an impediment to getting tested. A minority reported that the mobile clinic van was not the preferred place to receive HIV screening. It is also unknown what the acceptability of mobile POCT testing among a broader cross section of MSM in this regional community, or among MSM in other regional areas and represents a target for future research; as well as more detailed associations between prevalence, risk and previous testing. Further, additional evaluation is needed regarding sustainability and cost-effectiveness.

A mobile van clinic in a regional area is feasible, however it is resource- and time-intensive. In rural areas, people indicated a desire to test at home and would prefer that to a park. The peer-tester presence in this project aided in discussions and offers for opportunistic testing demonstrated acceptable health promotion, but not a sustainable testing option; further investigation into HIV self-testing is merited. There were many significant barriers to implementing the project such as weather, logistics and initial stakeholder engagement. Further the plan for a night clinic was changed shortly to an afternoon clinic due to safety concerns for the staff. Based on the documented field notes the peer-testers displayed resilience and remained proactive to ensure that the project continued.

### Recommendations

- A mobile van clinic is acceptable and feasible, however it is time- and resource-intensive.
- Maximising access to non-judgemental HIV testing may help to reduce barriers to access and address regional health disparities in Queensland populations
- HIV self-testing is acceptable and could more cost-effective for dispersed regional populations; and has recently received TGA approval in Australia which can enhance feasibility and strategy. Further investigation is warranted.
- Further investigation into barriers to HIV testing is merited (i.e. of those potential clients who chose not to access the mobile clinic van).
- Models of beat outreach are widely practiced across Australian non-governmental organisations (NGO), however further exploration in engaging beat users to test for HIV is merited.

### Recommendations for the future

The project highlighted several strengths and achievements, and barriers to engagement and lessons learned. The field notes detailed several ideas concerning future recommendations for similar public health promotion initiatives. One key recommendation reflected the importance and challenge of promoting to the community that testing facilities exist that are confidential and accurate.

Similarly, recommendations were raised that increased the potential scope of testing to include other STIs, which would engage people more widely with the concept of testing. It was noted that other organisations had sexual health poster promotions in public places (e.g., toilet stalls) and partnering with these local organisations may be a way to generate greater awareness and acceptance within the target group, and ultimately promote more testing and more resource-effective health promotion [37].

## Conclusions

POCT recruitment was initially slow but grew over time as potential clients saw benefits of accessing testing through the mobile clinic. It was the hope of the health promotion to encourage HIV testing and serve as a snowball approach for (unmonitored) beat communication. Recruiting MSM via geosocial apps in a regional area was found to be acceptable and feasible. Future research is warranted to investigate alternative methods of engaging regional MSM (e.g., co-location of POCT with an existing trusted regional service) and consideration of implementing a HIV self-testing kit programs in regional populations, secondary to recent TGA approval in Australia and greater investment to support regional areas.

## List of Abbreviations

- Human Immunodeficiency Virus (HIV) - Virus that attacks the immune system by destroying white blood cells needed for the body’s defence against viruses.
- Men who have sex with men (MSM) - Refers to men who engage in a form of sexual intercourse with men.
- People Living with HIV (PLHIV) - Refers to those who have received a notification of a positive HIV/AIDS status.
- Point of Care Testing (PoCT) - Health testing that is performed on site, often with results available during the visit.
- Post Exposure Prophylaxis (PEP) - A four-week course of medication commenced within 72 h of suspected or confirmed exposure to HIV.
- Pre-exposure prophylaxis (PrEP) - Daily medication that prevents a person who has been exposed to HIV from acquiring the virus by blocking the enzyme that allows HIV to reproduce in the human body.
- Rapid testing - Quick, on the spot testing for HIV in which results are provided are on site.
- Sex on premises venues (SOPV) - Refers to commercial venues in which patrons meet to engage in sexual activity, in this study it refers to Club 29 which is a male only venue.
- Sexually transmitted infection (STI) - Infections that are spread through sexual contact. This study tested for Syphilis, Chlamydia and Gonorrhoea specifically.

## Additional file

Additional file 1: The post-test questionnaire used in this study was created by Queensland Positive People as per the organisation’s standard evaluation procedure with additional items included that were relevant to the current project and research questions. This questionnaire has been provided in a supplementary file and included in submission. (DOCX 81 kb)
