# Supplementary material for: Point-of-care testing (POCT) for HIV/STI targeting MSM in regional Australia at community ‘beat’ locations
Source: BMC Health Serv Res. 2019 Feb 2;19:93. doi: 10.1186/s12913-019-3899-2 (PMC6359847; doi:10.1186/s12913-019-3899-2)
Supplement: Supplementary file 1 — The post-test questionnaire used in this study was created by Queensland Positive People as per the organisation’s standard evaluation procedure with additional items included that were relevant to the current project and research questions. This questionnaire has been provided in a supplementary file and included in submission. (DOCX 81 kb) [file 12913_2019_3899_MOESM1_ESM.docx]

*Additional Files*

The post-test questionnaire used in this study was created by Queensland Positive People as per the organisation’s standard evaluation procedure with additional items included that were relevant to the current project and research questions.

| **Toowoomba Survey** | | | | | | | | | | **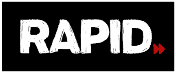 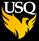** | | |
| --- | --- | --- | --- | --- | --- | --- | --- | --- | --- | --- | --- | --- |
| **Age:** | | | | **Postcode:** | | | **Country of Birth:** | | | | | |
| **Indigenous Status:** | | Aboriginal  Torres Strait Islander  Aboriginal and Torres Strait Islander  Neither | | | | | **Testing History:** | My last HIV test was less than 12 months ago  My last HIV test was over 12 months ago  I have never tested for HIV | | | | |
| **Gender:** | Male  Female  Trans* | | **Do you have** Yes  **a Medicare**  No  **Card?** | | | **I have sex with:** | | Men  Women  All genders | **I inject:** | | | Nothing  Drugs  Steroids |
| **How did you hear about RAPID?** |  | | I received a coupon  I’ve been to RAPID before | | Grindr  Recommendation/ Word of Mouth | | | Squirt  Other *(please specify)*: | | |  |  |

|  | Strongly Agree | Agree | Not Sure | Disagree | Strongly Disagree | Not Applicable |
| --- | --- | --- | --- | --- | --- | --- |
| A peer-led, community based testing service like the RAPID mobile van clinic would increase my HIV testing frequency | 🞏 | 🞏 | 🞏 | 🞏 | 🞏 | 🞏 |
| I would be happy to refer a friend for HIV/sexual health testing via an incentive coupon | 🞏 | 🞏 | 🞏 | 🞏 | 🞏 | 🞏 |
| I would have had a HIV test regardless of whether the mobile clinic van was available | 🞏 | 🞏 | 🞏 | 🞏 | 🞏 | 🞏 |
| *Where?* |  |  |  |  |  |  |
| Community HIV testing from a mobile clinic van is an acceptable HIV testing method | 🞏 | 🞏 | 🞏 | 🞏 | 🞏 | 🞏 |
| *Why/Why not?* |  |  |  |  |  |  |
| I find it easier to test for HIV from a mobile clinic van located near a ‘beat’ | 🞏 | 🞏 | 🞏 | 🞏 | 🞏 | 🞏 |
| If the mobile clinic van came regularly, my HIV/sexual health testing frequency would increase | 🞏 | 🞏 | 🞏 | 🞏 | 🞏 | 🞏 |
| I would prefer to test for HIV anonymously | 🞏 | 🞏 | 🞏 | 🞏 | 🞏 | 🞏 |
| I would be willing to use a HIV home testing kit after a referral from the mobile clinic van | 🞏 | 🞏 | 🞏 | 🞏 | 🞏 | 🞏 |
| I would be willing to use a HIV home testing kit in the future | 🞏 | 🞏 | 🞏 | 🞏 | 🞏 | 🞏 |
| *What types of support would be helpful if using HIV home testing?* |  |  |  |  |  |  |
| I would be willing to pay for a HIV home testing kit | 🞏 | 🞏 | 🞏 | 🞏 | 🞏 | 🞏 |
| *How much? (please circle)* | $0 -$10 | $10- $20 | $20- $30 | $30-  $40 | $40-  $50 | $50+ |
| Do you experience any barriers to HIV testing in Toowoomba? | | | | | | |
